# Inflammation, diabetic foot and related treatments

**DOI:** 10.3389/fendo.2025.1676621

**Published:** 2025-10-07

**Authors:** Yuqi Qin, Shan Deng

**Affiliations:** ^1^ Liyuan Hospital, Tongji Medical College, Huazhong University of Science and Technology, Wuhan, China; ^2^ Department of Cardiovascular Medicine, Union Hospital, Tongji Medical College, Huazhong University of Science and Technology, Wuhan, China

**Keywords:** diabetic foot, inflammatory regulatory network, NF-κB signaling pathway, interleukin family, targeted therapy

## Abstract

Diabetic foot, one of the most severe chronic complications of diabetes mellitus, arises from complex interactions among neuropathy, vascular ischemia, and inflammatory dysregulation. This review systematically explores the inflammatory regulatory network centered on NF-κB and the interleukin (IL) family in the diabetic foot microenvironment and examines their roles in promoting tissue damage. Under hyperglycemic conditions, the AGE-RAGE axis potently activates the NF-κB signaling pathway, leading to upregulation of pro-inflammatory factors and suppression of anti-inflammatory mediators, thereby forming a vicious cycle of “inflammation-oxidative stress-tissue damage.” Charcot foot, a distinct subtype characterized by neurogenic osteoarthropathy, is strongly linked to abnormal IL-6/RANKL pathway activation and impaired anti-inflammatory neuropeptide signaling. We further assess the diagnostic utility of inflammatory biomarkers, including procalcitonin (PCT), high-sensitivity C-reactive protein (hs-CRP), and the neutrophil-to-lymphocyte ratio (NLR), and summarize current therapeutic challenges along with emerging targeted strategies, such as NF-κB inhibition via salicylates. This review underscores the importance of spatiotemporal heterogeneity and dynamic equilibrium within inflammatory networks for clinical stratification and proposes that integrating multi-omics data with artificial intelligence models holds promise for developing personalized interventions. This approach offers novel theoretical insights to overcome therapeutic bottlenecks in diabetic foot management.

## 1 Background

### 1.1 Epidemiology and global burden of diabetic foot

Diabetes represents a major global public health challenge. It is projected that by 2040, the global number of people with diabetes will increase to 13.5–17.4 million (1). Its complications significantly compromise patient quality of life. Diabetic foot, in particular, has garnered significant clinical concern due to its association with high disability rates and substantial healthcare burden. Non-healing ulcers in diabetic foot patients frequently result in amputation, with over 100,000 such procedures performed annually for refractory diabetic foot wounds (2). A prospective study demonstrated that patients with uncontrolled diabetes exhibit a 7.25-fold higher risk of postoperative complications following foot surgery relative to non-diabetic individuals (3).

### 1.2 Pathophysiology of diabetic foot: a multifactorial disorder

The pathological mechanism of diabetic foot is notoriously complex, entailing interactions between neuropathy, vascular ischemia, and immune dysregulation. Traditionally, research focused on sensory loss and foot deformities secondary to neuropathy. Contemporary studies, however, emphasize the central role of inflammatory regulatory networks. As a core pathological driver, sustained inflammatory responses activate signaling pathways including NF-κB and interleukin families, thereby exacerbating oxidative stress. This induces reactive oxygen species (ROS) accumulation, leading to lipid peroxidation and glycation reactions that generate both advanced lipoxidation end products (ALE) and advanced glycation end products (AGE) (4). Excessive ALE and AGE can cause protein cross-linking and aggregation, altering cell signal transduction and function, resulting in tissue damage. Tissue damage further triggers the inflammatory response, forming a vicious cycle of “inflammation-oxidative stress-tissue damage”. In a high-glucose environment, the body activates NF-κB through the AGE-RAGE axis, upregulating the expression of pro-inflammatory factors such as IL-6 and TNF-α, and simultaneously inhibiting the secretion of anti-inflammatory mediators such as IL-10D, ultimately leading to an imbalance between pro-inflammatory and anti-inflammatory factors (5, 6). This imbalanced state not only increases the risk of ulcer infection but is also associated with special complications such as Charcot foot. Charcot foot is typically characterized by neurogenic bone destruction. In its acute phase, the abnormal activation of the IL-6/RANKL pathway accelerates bone resorption. At the same time, the deficiency of anti-inflammatory neurotransmitters further disrupts the homeostasis of bone metabolism (7–10).

### 1.3 Current management strategies and unmet needs

Diabetic foot is prone to misdiagnosis or missed diagnosis in the early stage, and the possible causes are as follows: (1) Insufficient awareness of non-diabetic lower limb ischemic diseases among some primary care physicians: They have difficulty distinguishing diabetic foot from lower extremity arteriosclerosis obliterans and thromboangiitis obliterans. (2) Limitations and insufficient accessibility of imaging examination techniques: X-rays cannot detect early infection or microcirculation disorders. Although magnetic resonance imaging (MRI) has high soft tissue resolution, it is expensive and difficult to be popularized in primary medical institutions. (3) Need for comprehensive assessment by a multidisciplinary team for diabetic foot diagnosis: The diagnosis of diabetic foot requires the comprehensive evaluation of a multidisciplinary team (e.g., endocrinology department, vascular surgery department), and diagnostic deviations are likely to occur due to incomplete information. (4) Insufficient disease awareness and weak self-management awareness of some diabetic foot patients: Due to neuropathy-induced reduced foot sensation, they ignore minor injuries, resulting in delayed diagnosis and treatment (11, 12).

The current standard management of diabetic foot mainly includes blood glucose control, vascular assessment, wound decompression, surgical debridement, and topical dressings. The wound healing of patients with diabetic foot is positively correlated with good blood glucose control (13). Existing studies have shown that maintaining glycated hemoglobin at 7.0%-8.0% during treatment can promote the wound recovery of patients with diabetic foot ulcer (DFU) without increasing the mortality rate (14). The most common cause of diabetic foot is that the hypoesthetic foot bears excessive mechanical pressure. Reducing excessive mechanical stress on the sole of the foot is of great significance for preventing diabetic foot ulcers (13), such as bed rest, wheelchairs, and surgical decompression (15). However, various decompression devices such as decompression shoes and removable cast walkers have not been fully utilized in clinical practice. Advanced technologies such as vacuum sealing drainage and ultrasonic debridement are mainly concentrated in tertiary hospitals, making it difficult for patients in rural or remote areas to access them. When there is necrosis, infection, or abscess in the wound of patients with diabetic foot, surgical debridement is an effective and preferred method to avoid further deterioration of the wound (16). At present, the treatment of diabetic foot faces two major dilemmas: a relatively high rate of surgical complications and insufficient targeted therapies. Although basic research has shown that the regulation of NF-κB or the IL family can improve wound healing (10, 17, 18), new breakthroughs are still needed in practical translational applications.

### 1.4 Objective and scope of this review

This study focuses on the inflammatory regulatory network and systematically analyzes the spatio-temporal expression characteristics of inflammatory markers such as NF-κB, the IL family, procalcitonin, and high-sensitivity C-reactive protein in the microenvironment of diabetic foot. The purpose of this study is to reveal the interaction mechanism of these markers, providing a scientific theoretical basis for the development of precise intervention strategies. This exploration not only helps to break through the existing treatment bottleneck but also has extremely important clinical significance for improving the prognosis of patients and reducing the medical burden. Despite advances in conventional management, there is still a lack of targeted anti-inflammatory therapies for diabetic foot ulcers. Understanding the interplay of inflammatory mediators could open new avenues for precision treatment.

## 2 A special type and classification of diabetic foot

### 2.1 Charcot foot

#### 2.1.1 Epidemiology and clinical burden

Charcot foot is a rare but severe complication of diabetes. Research investigations have shown that in Denmark, the incidence of Charcot foot in the general population is 7.4 cases per 10,000 person-years, and the prevalence of Charcot foot among diabetic patients is 0.56% (8). It is an inflammatory lesion that occurs in patients with peripheral neuropathy. The lesion involves the bones, joints, and soft tissues of the foot and ankle. Currently, the most common underlying cause is diabetes (19).

#### 2.1.2 Clinical features and diagnosis

The occurrence and progression of diabetic Charcot foot result from the synergistic interaction between neuropathy and inflammation, which collectively impair the stability of the bone-joint structure and trigger pathological processes. Neuropathy affects bone integrity, joint sensation, and blood flow regulation in the distal extremities, leading to the loss of protective sensation and autonomic nerve dysfunction. Inflammation, on the other hand, exacerbates tissue damage through mechanisms such as immune cell activation and cytokine release (20). Acute local inflammation of the foot and ankle can lead to varying degrees of destruction, subluxation, dislocation, and foot deformities, including the typical “rocker-bottom” deformity caused by midfoot collapse (9). However, clinically, Charcot foot presents with atypical clinical manifestations, making it prone to misdiagnosis and missed diagnosis. Due to peripheral neuropathy, patients often have no obvious pain or only mild symptoms, and the condition is mostly unilateral. In the early stage, it may only present as foot redness, swelling, and increased skin temperature (more than 2°C higher than the contralateral side), which is easily mistaken for ordinary swelling or infection. Additionally, approximately 30% of patients show no abnormalities on X-rays at the initial diagnosis, and thus advanced imaging examinations such as magnetic resonance imaging (MRI) are required to confirm bone lesions. Charcot foot should be mainly differentiated from common infections, gout, deep vein thrombosis (DVT), and other conditions. Patients with common skin and foot infections may have ulcers and purulent secretions, along with significant increases in white blood cell (WBC) count and C-reactive protein (CRP) level; abscesses or sinuses can be detected on MRI. Gout typically occurs as acute attacks, accompanied by elevated serum uric acid levels, and tophi can be observed on X-ray examinations. The main manifestation of DVT is symmetrical lower limb swelling; ultrasound can detect thrombi but no bone lesions. Clinicians need to diagnose Charcot foot through a multimodal verification model that closely integrates the patient’s clinical manifestations, hierarchical imaging examinations (e.g., X-ray for screening fractures and dislocations, MRI for detecting bone marrow edema and soft tissue inflammation), and laboratory tests to rule out other diseases. This approach is essential to avoid missed diagnosis and misdiagnosis (21).

#### 2.1.3 Pathophysiology: neuropathy and inflammation

Charcot foot can be divided into active and inactive phases, as well as acute and chronic stages. In the acute stage of Charcot foot, generally in febrile patients with normal vital signs and normal blood infection indicators, the foot is characterized by swelling, heat, and erythema. Usually, compared with the contralateral side, the skin temperature increases (ranging from 2 °C to 8 °C). Due to diabetic peripheral neuropathy, patients have decreased or absent foot sensation and do not necessarily experience strong pain, which may be related to impaired deep tendon reflexes, especially the Achilles tendon reflex (22, 23). This stage is usually undiagnosed and progresses rapidly, eventually developing into the chronic stage, accompanied by severe deformities, bone prominences, and foot structure deformities (24).

**Figure 1:**
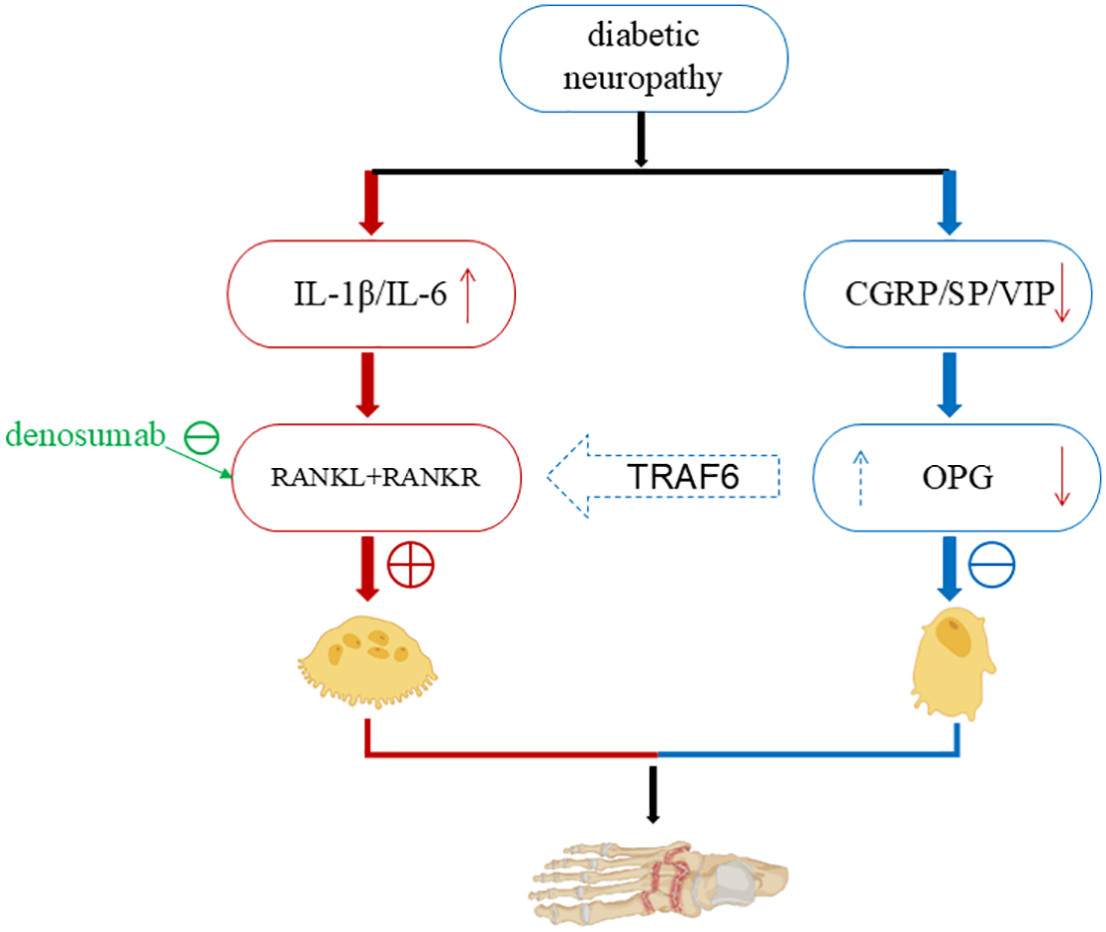
The relationship between inflammation and neuropathy in Charcot foot. When Charcot foot is in the inflammatory phase, local T helper cells and macrophages can activate a variety of pro - inflammatory factors, including IL-1β, IL-6, NF-κB, etc., which stimulate the maturation of osteoclasts (25) (left). While pro-inflammatory factors stimulate the generation and resorption of osteoclasts, anti - inflammatory agents (such as the neurotransmitter Substance P, calcitonin gene - related peptide, vasoactive intestinal peptide) promote the maturation of osteoblasts (right). At the same time, they inhibit the over - activity of osteoclasts through TRAF6. The pro - inflammatory process is balanced with the anti - inflammatory process, the damaged bone is removed, the remaining bone is reconstructed, and the restoration and replacement of tissues are initiated. When neuropathy occurs in the foot, the neuropathy leads to the deficiency of CGRP, SP and nerve growth factor, which further reduces the synthesis of the RANKL antagonist OPG (28–30). Then the pro - inflammatory process dominates, osteoclasts become active, causing excessive bone destruction and aggravating inflammation and ulcers in the foot. (Created with BioRender.com).

When Charcot foot is in the inflammatory stage, local T helper cells and macrophages can activate various pro-inflammatory factors, including IL-1β, IL-6, NF-κB, etc. Part of their role is to stimulate the maturation of osteoclasts, thereby removing damaged bone tissue so that the remaining bone can be reconstructed. The inflammatory process of Charcot foot is closely related to neuropathy. Pro-inflammatory factors stimulate osteoclast formation and resorption, while anti-inflammatory agents [such as the neurotransmitter substance P, calcitonin gene-related peptide (CGRP), and vasoactive intestinal peptide (VIP)] promote osteoblast maturation (**Figure 1**). Animal studies have shown that the neurotransmitter CGRP, which can promote the release of anti-inflammatory factors, can activate osteogenesis (25). The pro-inflammatory process needs to be balanced with the anti-inflammatory process to avoid excessive tissue damage while initiating tissue recovery and replacement. The imbalance between the pro-inflammatory and anti-inflammatory processes is a major characteristic of Charcot foot disease.

Patients with existing foot neuropathy have reduced or absent release of anti-inflammatory neurotransmitters. If the foot lesion experiences repeated trauma or persistent infection, the balance between pro-inflammatory and anti-inflammatory is disrupted, and the pro-inflammatory state dominates, resulting in persistent foot damage and inflammation. Chen et al. demonstrated that nerve conduction velocity (NCV) is positively correlated with total hip bone mineral density (BMD) in male patients with type 2 diabetes mellitus (T2DM). A decrease in NCV indicates an increased risk of osteopenia in these patients (26). Another prospective study involving 1,602 diabetic patients with Charcot foot also showed that, compared with diabetic patients without Charcot foot, those with Charcot foot have a higher risk of fractures (including foot fractures, lower leg fractures, etc.) and osteoporosis (27).

#### 2.1.4 Bone remodeling and the RANKL pathway

Bone remodeling is coordinated by the activities of osteoblasts (OB) and osteoclasts (OC). OBs are derived from mesenchymal stem cell precursors, and OCs are derived from hematopoietic precursors. The RANKL-RANK signaling pathway is crucial for the occurrence and differentiation of OCs. RANKL can activate the RANK receptor of OC precursors, and OPG can block its action. Intracellular signal transduction is mediated by TRAF6. Related transcription factors such as c-Fos and NFATc1 are involved in OC development. In patients with Charcot foot, there is increased inflammatory activity and bone resorption, elevated serum inflammatory markers and RANKL levels. IL-1β and IL-6 induce excessive production of RANKL, and OC activity increases in bone samples. Allele loci in patients with Charcot foot, such as certain allele polymorphisms of RANKL and OPG, increase the likelihood of Charcot foot occurrence. An increased RANKL/OPG ratio in the blood is associated with neuropathy and the risk of developing Charcot foot disease (10). The deficiency of CGRP, SP, and nerve growth factor (NGF) caused by neuropathy can reduce the synthesis of osteoprotegerin (OPG), an antagonist of receptor activator of nuclear factor-κB ligand (RANKL) (28–30), further exacerbating foot inflammation and ulcers.

#### 2.1.5 Therapeutic advances and future directions

Recently, with the description of the long-term activation of the RANKL - NF-κB pathway, there has been great interest in the potential of using denosumab to treat acute Charcot foot. Denosumab is a fully monoclonal anti-RANKL antibody that binds to and inhibits RANKL, reducing bone resorption by inhibiting the recruitment, maturation, and action of osteoclasts. Recent observational studies have reported that denosumab may be an effective drug for treating acute Charcot foot, shortening the treatment time, but there is a lack of randomized controlled trial data (31, 32). Shofler et al. administered a single subcutaneous injection of 60 mg denosumab to 7 patients with acute Charcot neuroarthropathy, combined with total contact casting (TCC), weight-bearing restriction, and follow-up visits every two weeks until the skin temperature returned to normal, with a total follow-up duration of 1 year. Among these patients, one experienced transient muscle pain in the upper limb on the injection side (relieved after 1 month), and another developed a diabetic foot infection in the contralateral lower limb during the 6–9 month follow-up period (cured after oral antibiotic treatment); the remaining patients showed good tolerance to the drug (33). However, a review on the discontinuation of denosumab therapy for osteoporosis concluded that discontinuation may lead to a rapid rebound in bone turnover, loss of bone mineral density (BMD), and an increased risk of fractures—particularly in patients who received long-term denosumab treatment (34). Other drugs used for the treatment of Charcot neuroarthropathy but still in the clinical trial phase include tumor necrosis factor-alpha (TNF-α) inhibitors, vitamin D analogs, and interleukin-6 (IL-6) inhibitors (21).

However, most diabetic patients with neuropathy do not develop active Charcot foot, which may be related to the limited vasodilatory ability caused by neuropathy. Peripheral neuropathy leads to reduced release of the neurotransmitter nitric oxide (NO), limiting the vasodilatory ability of neuropathic blood vessels. Nitric oxide is both pro-inflammatory and an effective vasodilator. In diabetic oxidative stress, the reduced release of nitric oxide increases the activity of vascular matrix metalloproteinases, thereby inhibiting vasodilation (35, 36).

## 3 The relationship between inflammation and diabetic foot and the possible pathogenesis

### 3.1 Diabetic foot and NF-κB

**Figure 2:**
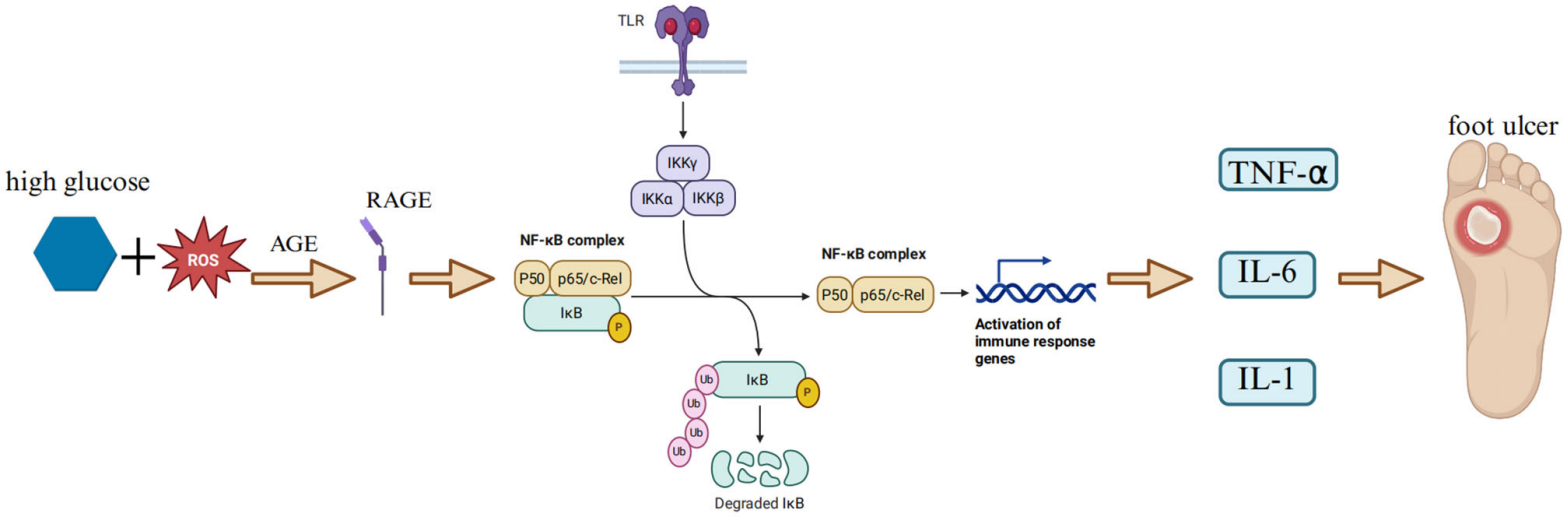
NF-κB - cytokine pathway. In a hyperglycemic environment, oxidative stress triggers lipid peroxidation and glucose oxidation reactions, leading to the formation of highly reactive and electrophilic compounds. These compounds attack the free amino groups in proteins, causing covalent modifications and subsequently generating advanced lipoxidation end - products (ALE) and advanced glycation end - products (AGE) (4). AGE - RAGE signal transduction promotes the activation of NF – κB (35). When cells are stimulated, the IKK complex (including IKKα and IKKβ) phosphorylates IκBα. Subsequently, IκBα undergoes ubiquitination and degradation, releasing the transcription - activating subunits of NF - κB. These subunits (such as p65 - p50 and RelB - p52 heterodimers) enter the nucleus, recruit transcriptional co - activators (such as CREB - binding protein CBP/p300 and histone acetyltransferase HAT), and bind to target DNA elements to activate the transcription of downstream molecules (47), promoting the expression of pro - inflammatory enzymes and pro - inflammatory factors, such as IL - 6 and TNF – α (51), which further exacerbates wound inflammation. Pro - inflammatory cytokines such as IL - 1β and TNF - α are classical activators of NF - κB, and the activation of NF - κB further aggravates oxidative stress (36), thus forming a vicious cycle of inflammation - oxidative stress - tissue damage. (Created with BioRender.com).

As a redox-sensitive transcription factor, NF-κB can be activated by various stimulating factors, including hyperglycemia, oxidative stress, and pro-inflammatory cytokines (37–39). Oxidative stress originates from the utilization of oxygen during aerobic respiration in organisms. It is manifested as a persistent imbalance between the production of reactive oxygen species (ROS) and the detoxification ability of the endogenous antioxidant system (AOS) (40). Oxidative stress can trigger lipid peroxidation and sugar oxidation reactions, promoting the formation of highly reactive and electrophilic compounds. These compounds can attack the free amino groups in proteins, causing covalent modifications and subsequently generating advanced lipoxidation end products and advanced glycation end products (AGE) (4). In oxidative stress, the NF-κB signaling pathway is the main switch system that activates pro-inflammatory and pro-oxidative responses (41). It is believed that the overexpression of the NF-κB gene or protein can lead to enhanced oxidative stress, thereby affecting wound healing in diabetic patients (42). In a streptozotocin (STZ)-induced diabetic mouse model, ischemia-reperfusion injury induced the overexpression of NF-κB in sciatic endothelial cells and Schwann cells of diabetic mice. Compared with the control group, there was an increased expression of ICAM-1 and extensive infiltration of mononuclear macrophages, suggesting that the enhanced diabetic neuroinflammatory response was mediated by NF-κB activation (43). In recent years, studies have found that miR-19a/b and miR-20a can reduce toll-like receptor 3 (TLR3)-mediated nuclear factor kappa B (NF-κB) activation (characterized by suppressed phosphorylation and nuclear translocation of p65), thereby decreasing the production of inflammatory chemokines (e.g., CXCL5, interleukin-8 [IL-8]) and cytokines, limiting the infiltration of immune cells such as neutrophils, and promoting the resolution of wound inflammation and wound healing (44). Oxidation-related stress-induced AGE-RAGE signal transduction promotes NF-κB activation, leading to diabetic vascular inflammatory injury (40). AGE can bind to the receptor RAGE, greatly accelerating the progression of the disease and the development of its microvascular complications, such as diabetic nephropathy, retinopathy, and neuropathy (45–48). Capillary damage, thickening of the capillary basement membrane, increased vascular permeability, and disruption of the blood-tissue barrier, as well as the increased adhesion of leukocytes to endothelial cells and neovascularization observed in experimental animal models of diabetes, are all related to the AGE-RAGE axis (49). Research has shown that the receptor for advanced glycation end-products (RAGE) is a receptor associated with the persistent activation of NF-κB in the diabetic microenvironment and is central to sensory neuron dysfunction. During sural nerve biopsy, the ligand of RAGE and the receptor itself co-localized NF-κBp65 and IL-6 in the microvasculature of patients with diabetic neuropathy. In the peripheral nerves of diabetic mice, advanced glycation end products induced the activation and upregulation of NF-κB and NF-κB-dependent gene expression, which was inhibited by RAGE blockade. An important feature of RAGE-mediated NF-κB activation is that it upregulates the expression of NF-κBp65, thereby promoting the continuous activation of this transcription factor in an environment rich in RAGE ligands (50). The activation process of NF-κB involves the classical pathway and the non-classical pathway. Under normal conditions, NF-κB is retained in the cytoplasm by directly interacting with inhibitory proteins (such as IκBα). The classical pathway is usually triggered by IL-1 receptor (IL-1R), TNF receptor (TNFR), and pattern recognition receptors (PRRs), and is achieved through the activation of downstream IKKβ and IκBα and the release of p65-p50 (51). The non-classical NF-κB pathway is usually activated by CD40 ligand and lymphotoxin-β and depends on the activation of IKKα and the release of p52-RelB. In addition, phosphorylation of specific phosphorylation sites on the NF-κB p65 subunit leads to the selective transcription of downstream pro-inflammatory genes (52) (**Figure 2**). Previous studies have shown that Adenovirus 7 can stimulate NF-κB located in the cytoplasm. Subsequently, IκB is degraded, and NF-κB is phosphorylated and activated. The phosphorylated NF-κB translocates into the nucleus and binds to the IL-6 promoter, inducing the upregulation of IL-6 expression (53). Pro-inflammatory cytokines such as IL-1β and TNF-α are classical activators of NF-κB. They are produced in the early development of DR and have an important impact on the activation of NF-κB. These cytokines show strong activation effects on all tested p65 phosphorylation sites (54). The cytokine TNF-α derived from NF-κB can promote the overexpression of cyclooxygenase-2 (COX-2) and activate mitogen-activated protein kinase (MAPK) (55, 56). Both of these phenomena are related to the pro-inflammatory response and neuropathy caused by diabetes. For example, in two experimental models of T1DM neuropathy, our group found that the levels of TNF-α in the sciatic nerves of diabetic rats and mice were significantly increased, which was related to the dysfunction of large and small nerve fibers. This was evidenced by the decrease in the motor and sensory nerve conduction velocities and the intraepidermal nerve fiber density in diabetic animals (57). The NF-κB signaling pathway plays an important role in the healing process of diabetic foot ulcers (DFU). The NF-κB pathway regulates hundreds of genes, which are involved in many key cellular responses, including inflammation, cell migration, proliferation, and apoptosis (58). Specifically, NF-κB regulates the expression of pro-inflammatory genes in macrophages and granulation tissue and enhances the expression of degradative enzymes in cytokine synthesis, including cytokines, chemokines, and adhesion molecules (59). The activated NF-κB complex translocates into the nucleus, binds to DNA at the B-binding site, and promotes the expression of pro-inflammatory enzymes and pro-inflammatory factors, such as IL-6, tumor necrosis factor (TNF)-α, and inducible nitric oxide synthase (iNOS)(50]. In addition, NF-κB-mediated inflammatory disorders can cause wound inflammation and neurodegenerative diseases (60). High blood glucose levels can induce the activation of NF-κB, promote apoptosis of human endothelial cells, and delay wound healing in diabetic patients (61). Some studies have confirmed that negative pressure wound therapy can inhibit the release of pro-inflammatory enzymes and cytokines by preventing the activation of NF-κB, thereby increasing the wound healing rate, reducing the healing time, and decreasing the risk of amputation (62). Hyperbaric oxygen therapy can activate the NF-κB signal. This process can promote the expression of stromal cell-derived factor-1 and vascular endothelial growth factor in fibroblasts. In this way, it regulates cell proliferation and migration, thereby promoting angiogenesis and wound healing (63). The NF-κB signaling pathway is one of the key pathways for nuclear signal regulation. The functional changes in this pathway directly determine the wound healing situation in DFU. However, the key molecules that determine the activation or inhibition of the NF-κB signaling pathway are not yet clear, so in-depth research is needed.

### 3.2 Diabetic foot and interleukin family

The inflammatory milieu in diabetic foot ulcers (DFUs) is characterized by a complex network of cytokines and chemokines, driven by metabolic dysregulation and significantly influencing healing outcomes.

1. Cytokine and Chemokine Activation in DFU.

Glucotoxicity, insulin deficiency, and lipotoxicity can generate neuronal oxidative/nitrosative stress and activate various downstream kinases, such as protein kinase C (PKC), mitogen-activated protein kinase (MAPK), and c-Jun N-terminal kinase (JNK), as well as redox-sensitive transcription factors (such as NF-κB). These factors play a crucial role in triggering the cascade production of cytokines and chemokines. The cytokines involved include pro-inflammatory IL-1β, IL-2, IL-6, IL-8, and TNF-α. The chemokines involved include chemokine (C-C motif) ligand 2 (CCL2) and chemokine (C-X-C motif) ligand 1 (CXCL1) (5, 6).

This activation is evident in local tissue environments. Hanna et al. of diabetic foot obtained skin biopsy samples from 12 patients with type 2 diabetes and 5 non-diabetic orthopedic patients. The samples were stained with specific antibodies to detect the expression of various chemokines, cytokines, and growth factors. The results showed that in the keratinocytes at the edge of diabetic foot ulcers, the expression of transforming growth factor-β1 (TGF-β1), TGF-β1 receptor type I (TGFβRI), granulocyte-macrophage colony-stimulating factor (GM-CSF), and epidermal growth factor (EGF) was significantly increased; in dermal endothelial cells, the expression of monocyte chemoattractant protein-1 (MCP-1), GM-CSF, CXCR1, and TGFβRI was increased, while the expression of interleukin IL-10, IL-15, and TGF-β1 was decreased; factors such as IL-8, CCR2A, IL-10 receptor, GM-CSF receptor, platelet-derived growth factor and its receptor, vascular endothelial growth factor and its receptor type II, EGF receptor, insulin-like growth factor-1, and nitric oxide synthase-2 were not upregulated in both types of cells (64).

Furthermore, some studies have shown that the expression levels of B-cell activating factor (BAFF) and C-reactive protein in patients with diabetic foot are significantly increased. Moreover, the level of BAFF is positively correlated with TNF-α, interferon family cytokines (such as IFN-α2, IL-28A/IFN-λ2, IFN-γ), and IL-10 family cytokines (such as IL-19, IL-22, IL-26), and negatively correlated with the IL-6 receptor family (such as gp130/sIL-6Rβ), indicating that BAFF may be related to the inflammatory response and immune regulation of DFU (65).

### 3.2 Biomarker studies and predictive value for healing

The systemic and local cytokine profile holds significant predictive value for DFU healing. A recent prospective study (66)found that the clinical measurement of serum proteins in diabetic patients can be a successful strategy for guiding the clinical management of DFU. The researchers obtained serum samples from diabetic foot patients participating in the prospective study, divided these patients into a healing group and a non-healing group, and conducted follow-ups every two weeks before the ulcer healing of the patients or the 12-week withdrawal visit.

The results of the study suggested that the levels of IL-10, IL-4, IL-5, IL-6, IL-13, and interferon-γ were higher in the healing group, while the levels of Fractalkine, IL-8, and TNFα were higher in the non-healing group (**Table 1**). The level of IL-10 remained stable in the healing group. The results of the study indicated that IL-4, IL-5, IL-6, IL-13, IL-10, and IFN-γ can predict DFU healing. Among them, IL-10 can be used as a single biomarker to predict DFU healing in continuous measurements, while Fractalkine, IL-8, and TNFα are indicators for predicting non-healing of DFU.

**Table 1:** Summary of key cytokines as predictors of DFU healing outcomes.

Cytokine/chemokine	Association with healing	Potential role & comments
IL-10	Pro-healing	Key anti-inflammatory cytokine. Stable, elevated levels are a strong positive predictor. Potential as a standalone biomarker.
IL-4, IL-5, IL-13	Pro-healing	Associated with T-helper 2 (Th2) immune responses, which may promote resolution of inflammation and tissue repair.
IL-6	Pro-healing	Context-dependent. In this study, higher levels were associated with healing, may be involved in pro-repair signaling.
IFN-γ	Pro-healing	Often considered pro-inflammatory, but can also have immunostimulatory and antibacterial roles beneficial in healing.
Fractalkine (CX3CL1)	Non-healing	Chemokine that recruits cytotoxic lymphocytes; elevated levels may indicate persistent inflammation.
IL-8 (CXCL8)	Non-healing	Potent neutrophil chemoattractant; high levels suggest sustained neutrophilic inflammation and tissue damage.
TNF-α	Non-healing	Master pro-inflammatory cytokine; drives inflammation and is a clear marker of a non-healing microenvironment.

### 3.3 Knowledge gaps and future directions

All these studies indicate that the occurrence and development of diabetic foot are closely related to the interleukin family. However, the specific relationship between various interleukins and diabetic foot is still unclear. The dual roles of certain cytokines (e.g., IL-6), the spatial and temporal dynamics of their expression within the wound microenvironment, and their interaction with other immune mediators require further investigation to translate these findings into targeted therapeutic strategies.

#### 3.3.1 Diabetic foot and IL-1β

Accumulating evidence indicates that activation of the NOD-like receptor protein 3 (NLRP3) inflammasome in macrophages (MΦ) contributes to the sustained inflammatory response and impaired wound healing associated with diabetes. In diabetic wounds, neutrophil extracellular traps (NETs) released by excessively infiltrated neutrophils trigger NLRP3 inflammasome activation and subsequent interleukin-1β (IL-1β) release from MΦ. Furthermore, NETs upregulate NLRP3 and pro-IL-1β expression levels via activating the TLR-4/TLR-9/NF-κB signaling pathway. They also induce the generation of reactive oxygen species (ROS), which promotes the dissociation and interaction between NLRP3 and thioredoxin-interacting protein (TXNIP), thereby facilitating NLRP3 inflammasome assembly and activation. NET elimination promotes wound healing by reducing NLRP3 inflammasome activation and modulating the infiltration of innate immune cells into the wound bed (67). Interleukin-1β (IL-1β), a key pro-inflammatory cytokine released by neutrophils and monocytes/macrophages, typically exhibits a significant peak during the initial phase of inflammation. However, in diabetic patients, dysregulated inflammatory responses often prevent IL-1β from reaching the expected peak levels observed in normal inflammation. This aberrant IL-1β response impairs the timely initiation and effective control of the inflammatory process, compromising infection defense and tissue repair (68). This mechanistic insight is corroborated by clinical findings. A staged evaluation involving 38 diabetic foot ulcer (DFU) patients (stratified into healing and non-healing groups) and a control group demonstrated that (69): At treatment initiation: The healing group had significantly lower polymorphonuclear neutrophil (PMN) levels but significantly higher IL-1β levels compared to the control group; During treatment (weeks 2 and 4): The healing group exhibited a significant increase in PMN levels and a significant decrease in IL-1β levels. In contrast, the non-healing group displayed persistently low PMN levels (particularly later in treatment) and IL-1β levels that remained significantly lower throughout the treatment period. The study concluded that the non-healing group exhibited insufficient release of pro-inflammatory cytokines, notably IL-1β, likely linked to underlying PMN dysfunction. A longitudinal clinical study revealed that the mRNA and protein levels of interleukin-1β (IL-1β) in the skin tissues of patients with diabetic foot ulcers (DFUs) were significantly higher than those in healthy skin. IL-1β is a key effector molecule in the pyroptosis pathway of diabetic foot ulcers. After mice with perforin-2 (PFN2) knockout were infected with Staphylococcus aureus, the absent in melanoma 2 (AIM2) inflammasome was activated, which promoted the assembly of apoptosis-associated speck-like protein containing a CARD (ASC) pyroptosome and the activation of caspase-1. Subsequently, pro-IL-1β was cleaved into mature IL-1β and released, leading to persistent inflammatory responses and the inhibition of wound healing (70). Cumulatively, these findings indicate that NETs over-activate the NLRP3 inflammasome through dual pathways—TLR/NF-κB signaling and ROS generation—leading to dysregulated IL-1β release. Concurrently, inherent inflammation dysregulation in diabetes prevents IL-1β from achieving an effective peak during the early inflammatory phase or resolving appropriately later. Clinical evidence further confirms that impaired DFU healing is significantly associated with persistently low IL-1β levels (reflecting inadequate pro-inflammatory responses) and neutrophil dysfunction. Therefore, compromised production, release, and dynamic regulation of IL-1β represent a critical factor influencing the inflammatory trajectory and ultimate recovery of diabetic foot wounds.

#### 3.3.2 Diabetic foot, IL-4 and IL-13

Interleukin-4 (IL-4) is a type 2 cytokine that plays an important role in the immune system, especially in regulating immune responses and inflammatory processes. Studies have shown that IL-4 can inhibit the phosphorylation of NF-κB p65 induced by high glucose. In human retinal endothelial cells, IL-4 can inhibit the phosphorylation of 4 p65 sites; in Müller cells, it can inhibit the phosphorylation of 5 sites (54). This inhibitory effect of IL-4 leads to a decrease in IL-6, IL-8, and TNF-α (Human Retinal Endothelial Cells, HREC), as well as TNF-α, IFNγ, CXCL-11, and iNOS (Müller cells), indicating that IL-4 has a protective effect (71, 72). IL-4 exerts its effect by binding to specific receptors. These receptors include the type I receptor composed of IL-4Rα and the γc chain (common γ chain), and the type II receptor composed of IL-4Rα and IL-13Rα1. IL-4Rα can also bind to IL-13Rα2, which has a high affinity and can secrete and block excessive IL-13 (73–75). IL-4 is mainly expressed by hematopoietic cells, while IL-13Rα1 is mainly found on structural cells. IL-4 and IL-13 signal through IL-4Rα, leading to the phosphorylation of STAT 6. STAT 6-deficient animals usually mimic IL-4Rα deficiency. However, there are also other signaling pathways, including the activation of STAT 3 (76). IL-13α2, as the third high-affinity IL-13 receptor, can act as a decoy receptor to further regulate the contribution of IL-13 (77). A 2024 study, using *in vitro* and *in vivo* experiments as well as clinical samples, demonstrated that interleukin-13 receptor alpha 2 (IL-13Rα2) actively participates in the disease process by promoting M2 polarization of macrophages and the expression of C-C motif chemokine ligand 2 (CCL2), thereby exerting a pro-fibrotic effect in radiation-induced lung injury (RILI) (78). In tissue repair, IL-4 is considered an anti-inflammatory cytokine. The anti-inflammatory properties mediated by IL-4Rα signaling may be one of the most profound ways in which type 2 immunity contributes to tissue repair. IL-4Rα signaling promotes the formation of an anti-inflammatory and wound-healing environment. IL-13 plays an important role in maintaining and supporting the appropriate matrix structure, indicating that the purpose of IL-13 is to maintain or remodel the matrix structure after injury (79). IL-4 and IL-13 regulate the quantity and quality of the extracellular matrix (ECM) through IL-4Rα signaling in multiple cells. Among them, IL-13 is the main profibrotic cytokine. The roles of IL-4 and IL-13 in tissue repair include inhibiting the initial inflammatory response and regulating the modification, degradation, and reconstruction of the ECM to achieve effective repair (80–82).

#### 3.3.3 Diabetic foot and IL-6

IL-6 is a pleiotropic cytokine with complex roles in inflammation, metabolism, and tissue repair, exhibiting context-dependent effects in diabetic foot ulcer (DFU).

##### 3.3.3.1 Diagnostic and prognostic role of il-6 in diabetic foot

IL-6 is an indicator of the early response to inflammation and trauma. Clinical studies have consistently demonstrated its elevated levels in DFU patients compared to diabetic patients without foot complications (83). Its utility extends to differentiating infection status, with significantly higher IL-6 levels observed in infected ulcers compared to non-infected ones in type 2 diabetes patients (84). Furthermore, IL-6 possesses prognostic value for disease severity. Some researchers observed and followed up patients after diabetic foot amputation and found that the IL-6 concentration in the amputation group (20.8 ± 12.3 pg/ml) was significantly higher than that in the non-amputation group (9.9 ± 9.3 pg/ml) (85). Post-operatively, IL-6 dynamics can serve as a follow-up marker; a significant rise peaking at 3 days after tibial transverse transport surgery, followed by a decline, correlates with the acute inflammatory phase and subsequent initiation of healing processes (86).

##### 3.3.3.2 IL-6 signaling pathways and bone remodeling

The molecular mechanism of IL-6 in bone remodeling, particularly relevant to Charcot neuroarthropathy, is mediated through the JAK/STAT pathway. An *in vitro* experiment using mice to establish an IL-6-stimulated osteocyte model showed that with the increase in IL-6 concentration, the phosphorylation of JAK-2 protein in the IL-6 pathway increased, and the expression level of RANKL also increased, suggesting that IL-6 and its receptor can promote osteocytes to express RANKL and are closely related to the activation of JAK2 (87). After the IL-6 cytokine binds to the receptor, it promotes the dimerization of the gp130 receptor to form a homodimer. Subsequently, the respective bound JAKs approach each other, phosphorylating the tyrosine residues of both, thereby activating the JAK activity. The activated JAK can phosphorylate the tyrosine residue at the C-terminus of the STAT molecule, and the position of this residue is precisely the binding site of the signal transduction and transcription factor with an SH2 structure. Two phosphorylated STAT molecules form a dimer through their respective SH2 structures, and then are transported into the nucleus and bind to the regulatory sequence of specific genes to regulate the expression of related genes (88). In synovial cells of joints, the activation of the STAT3 protein in the IL-6 pathway can further promote the downstream expression of RANKL (89).

##### 3.3.3.3 Dual role of IL-6: pro-inflammatory vs. protective effects

The role of IL-6 is inherently dualistic. Traditionally considered a pro-inflammatory cytokine often released alongside TNF-α (90), it also demonstrates protective and restorative functions under specific conditions. Experimental studies in diabetic rats report that pharmacological doses of IL-6 can improve motor and sensory nerve conduction velocities, correct neuropathic pain, and increase nerve blood flow (55, 91, 92). In addition, interleukin-6 (IL-6) released during acute exercise enhances insulin sensitivity via the AMP-activated protein kinase (AMPK) pathway and promotes glucose transporter 4 (GLUT4)-mediated glucose uptake, which can reduce visceral fat and associated inflammation. When IL-6 binds to its membrane-bound receptor (mbIL-6Rα), it activates the glycoprotein 130 (gp130)-Janus kinase/signal transducer and activator of transcription 3 (JAK/STAT3) pathway. This signaling cascade is primarily present in tissues such as skeletal muscle and hepatocytes, and exerts anti-inflammatory and metabolic regulatory effects (93). However, the mechanistic evidence for these neuroprotective and metabolic benefits in human DFU remains limited and requires further validation.

##### 3.3.3.4 Angiogenic potential and translational implications

IL-6 also emerges as a pro-angiogenic factor with implications for wound healing. Previous studies have observed that IL-6 can induce tumor cells to produce VEGF. VEGF is a known angiogenesis-promoting factor that promotes the formation of new blood vessels by stimulating the proliferation and migration of vascular endothelial cells (94). IL-6 can bind to the IL-6 receptor on endothelial cells, activate the downstream signal transduction pathway, and directly promote angiogenesis; or it can indirectly promote angiogenesis by influencing macrophage polarization and promoting the formation of M2 macrophages (95, 96). The observed surgical-induced peak and subsequent decline of IL-6 following tibial transverse transport technique suggest a potential link between its spatiotemporal expression and the initiation of healing angiogenesis (93). Nonetheless, the precise role and therapeutic potential of IL-6 in DFU angiogenesis warrant confirmation through robust animal experimental studies.

In summary, IL-6 plays a multifaceted role in diabetic foot, serving as a valuable biomarker for diagnosis and prognosis, a key driver of inflammatory bone loss, and a potential mediator of nerve repair and angiogenesis (**Table 2**). Its functional duality underscores the importance of contextual interpretation. Targeting IL-6 signaling therapeutically would require precise spatiotemporal modulation to inhibit its detrimental effects while preserving or enhancing its beneficial roles.

**Table 2:** Summary of the dual roles and clinical significance of IL-6 in diabetic foot.

Role/context	Clinical/biological significance	Key findings/mechanisms	References
Diagnostic & Prognostic Marker	Elevated in DFU vs. diabetes alone; distinguishes infected from non-infected ulcers; predicts amputation risk.	Level in amputation group: 20.8 ± 12.3 pg/ml vs. non-amputation: 9.9 ± 9.3 pg/ml.	(83–85)
Pro-inflammatory & Bone Resorption	Drives inflammation and bone destruction in Charcot foot.	Activates JAK2/STAT3 pathway → upregulates RANKL → promotes osteoclastogenesis.	(86–88)
Protective & Metabolic Effects	May improve neuropathy and metabolic health in specific contexts.	Improves nerve conduction velocity, increases nerve blood flow in diabetic rats; enhances insulin sensitivity via AMPK/GLUT4 pathway.	(55, 90–92)
Angiogenic Potential	May promote blood vessel formation, crucial for wound healing.	Induces VEGF production; activates endothelial cells directly or via M2 macrophage polarization. Levels peak post-surgery (e.g., tibial transport).	(93–96)

#### 3.3.4 Diabetic foot and IL-8

Previous studies have shown that the activation of Toll-like receptor 9 (TLR9) induces bone marrow cells to release pro-inflammatory molecules, such as S100A8 and interleukin 8 (IL-8). This leads to the migration of bone marrow cells to the site of inflammation, which can affect the healing of diabetic foot ulcers and result in chronic inflammation (97). Some researchers conducted a biopsy analysis of the wounds of patients with diabetic feet and found that α-defensins and the inflammatory cytokine IL-8 were highly expressed. α-defensins and advanced glycation end-products (AGE) synergistically promoted the expression of IL-8, thus promoting the inflammatory response and leading to the formation of chronic wounds. The results of RT-PCR showed that advanced glycation end-products-bovine serum albumin (AGE-BSA) enhanced the expression of IL-8 in human foreskin fibroblasts (HFFs) in a time-dependent manner within 0–8 h and in a concentration-dependent manner within the range of 0–1 mg/mL (98). The biopsy results indicated that the expression of α-defensins was higher in the center of the wound than at the edge. α-defensins can upregulate the expression of IL-8, and IL-8 can guide neutrophils to reach the site of injury through the tissue matrix (99), indicating that many neutrophils accumulate at this site. IL-8 is a pro-inflammatory CXC chemokine, and it has been proven to promote cell proliferation, survival, and migration through autocrine activity (100). However, other studies have shown that the enhanced expression of IL-8 has been proven to induce the epithelial-mesenchymal transition (EMT) process in various human cancer cell lines, which is a physiological process in which epithelial cells acquire the motility characteristics of mesenchymal cells (101). An animal experiment showed that miR-203 (a microRNA that is particularly abundant in keratinocytes) was upregulated in the tissue of diabetic foot ulcers (DFUs), and it inhibited the epithelial-mesenchymal transition (EMT) process by targeting and inhibiting the phosphorylation of interleukin-8 (IL-8) and its downstream target AKT, thereby affecting the healing of DFU (102). The specific mechanism of the role of IL-8 in promoting chronic inflammation in diabetic foot ulcers is not yet clear. Whether it can promote the wound healing of patients with diabetic feet still needs to be verified by further animal experiments and clinical trials.

#### 3.3.5 Diabetic foot and IL-10

Recently, some researchers used cervical brushes to collect wound samples from patients with diabetic foot ulcers. The expression levels of the following cytokine genes were significantly increased in the collected samples: IL-1β (p = 0.0001), IL-6 (p = 0.0106), IL-10 (p = 0.0277), and TGFβ (p = 0.0002) (103). IL-10 is generally regarded as an anti-inflammatory cytokine. It can activate M2 macrophages and promote the wound healing process by inhibiting inflammation and stimulating the production of extracellular matrix (such as collagen) (104). IL-10 can inhibit the inflammation induced by IL-17. Therefore, the decrease in IL-10 content may promote the development of chronic non-healing foot ulcers. Some researchers isolated, cultured human placenta mesenchymal stem cells (PMSC) and implanted them into the wounds of a diabetic rat model. The experiment showed that the wounds in the PMSCs treatment group shrank significantly within 5 days, while the wounds in the control group healed slowly. ELISA analysis showed that after PMSC treatment, the local levels of pro-inflammatory cytokines TNF-α, IL-6, and IL-1 were significantly reduced, while the expression level of the anti-inflammatory cytokine IL-10 increased. To further confirm the role of IL-10 in wound healing, the researchers applied recombinant IL-10 to the wounds (subcutaneous injection, 100 ng per wound, starting on the second day and administered every other day until the end of the experiment). The results showed that the rIL-10 treatment enhanced wound healing, indicating that IL-10 plays a positive role in the healing of diabetic foot wounds (105).

#### 3.3.6 Diabetic foot and IL-17

IL-17 has been demonstrated to play a role in autoimmune diseases, such as inflammatory bowel disease (IBD), systemic lupus erythematosus (SLE), psoriasis, and ankylosing spondylitis (AS) (106). IL-17 is a pro-inflammatory cytokine released by T cells. IL-17 is highly expressed in pancreatic islets from donors with type 1 diabetes and type 2 diabetes, and it is mainly derived from β-cells and α-cells. This finding suggests that IL-17 may play a role in metabolic and immune stress, warranting further mechanistic investigations (107). In these studies, the high presence of IL-17 levels is associated with autoimmune diseases. A clinical study found that the expression levels of IL-17 were higher in patients with diabetes and diabetic foot compared to normal individuals, and it could be used as a diagnostic marker for diabetes and diabetic foot. Based on the results of this study, the authors pointed out that IL-17 was at a high level in the DFU and DM groups, probably because IL-17 belongs to the pro-inflammatory cytokines. For DFU patients, the high level of IL-17 in their bodies may stem from the inflammation at the ulcer site, the disruption of skin integrity, and various bacteria causing infections (108). IL-17 consists of six cytokines, IL-17 A - F; interleukin 25 (IL-25), also known as interleukin 17 E, is a unique member of the IL-17 family (109). Activated Th2 cells, eosinophils, mast cells, macrophages, epithelial cells, and endothelial cells can all express IL-25 (110, 111). IL-25 transduces signals through a heterodimeric receptor composed of IL-17RB and IL-17RA. According to relevant reports, it is involved in the process of tissue regeneration and also has regulatory effects on fibrosis and angiogenesis (99, 101, 102). Animal experiments have shown that local application of IL-25 to the wounds of diabetic mice can significantly improve wound healing, increase collagen deposition and angiogenesis. At the same time, IL-25 can reverse the downregulation of IL-17RB and β-catenin in diabetic wound tissues, improve the inhibitory state of the Wnt/β-catenin signaling pathway, and activate the AKT and ERK 1/2 signaling pathways, improve endothelial cell function, promote angiogenesis and cell migration, thus promoting the healing of diabetic wounds, suggesting that IL-25 may become a new target for the treatment of difficult-to-heal diabetic wounds (112). Researchers created full-thickness wounds on the dorsal surface of mice and treated them with anti-IL-17A, anti-IL-23, or isotype control antibodies. They found that IL-17 expression in the wounds of IL-23-deficient mice was significantly reduced, while this was not the case in IL-12-deficient mice. IL-17 expression could be significantly restored by injection of recombinant IL-23. IL-23 and IL-17-deficient mice showed a significant increase in non-inflammatory macrophages. In obese diabetic mice, wound re-epithelialization was significantly improved after treatment with anti-IL-17A and anti-IL-23p19 blocking antibodies. The study results indicate that by inhibiting IL-23 and IL-17, the healing process of diabetic wounds can be accelerated (113).

#### 3.3.7 Diabetic foot and IL-18

IL-18 is a pro-inflammatory cytokine involved in apoptosis and tissue damage (114). Some studies have shown that IL-18 is significantly elevated in the plasma of diabetic patients, increases with the quartiles of the insulin resistance index, and remains significant after adjusting for confounding factors (115). A case-control study found that monitoring the concentrations of IL-18 and IL-6 in the serum of type 2 diabetes patients has important clinical significance for the early prevention of diabetic foot and guiding anti-inflammatory treatment. The results of this study showed that the levels of IL-18, IL-6, white blood cells, and neutrophils in the diabetic foot group were significantly higher than those in the T2DM group. The results of univariate Logistic regression analysis showed that neutrophils, IL-18, and IL-6 were risk factors for the occurrence of diabetic foot. Further multivariate Logistic regression analysis indicated that IL-18 and IL-6 were independent risk factors for the occurrence of diabetic foot (116). The specific mechanism of the effect of IL-18 on diabetic foot is still unclear and awaits further research.

#### 3.3.8 Diabetic foot and IL-22

IL-22 is a cytokine whose functions involve the regulation of inflammatory responses, angiogenesis, neuronal differentiation, fibroblast growth, and re-epithelialization. These functions all play a role in the process of wound healing. However, to date, there are no definite research findings on the mechanism of action of IL-22 and its receptor in diabetic chronic skin ulcers, and further exploration and verification are still needed. Zeng et al. (117) conducted a study in which they selected 9 patients with T2DM and obtained the skin at the edge of their foot ulcers as the diabetic chronic skin ulcer group (DU group). At the same time, 12 patients with non-diabetic chronic skin ulcers (designated as the NDU group) and 10 normal skin tissues from the lower extremities remaining after orthopedic surgeries (designated as the NC group) were selected. The research results showed that in the NC group, IL-22 and IL-22R1 showed positive or even strongly positive expression, and their expression was mainly located in the cytoplasm of epidermal stratum corneum cells, dermal skin appendages, and superficial dermal nerve fibers. In the NDU group, IL-22 showed negative expression, while IL-22R1 showed weakly positive and positive expression. In the DU group, IL-22 showed weakly positive and positive expression, mainly located in the cytoplasm of epidermal stratum corneum cells, granular layer cells, and spinous layer cells, and IL-22R1 showed weakly positive expression. IL-22 is crucial for maintaining the integrity of the skin barrier and preventing subsequent infections. It can guide the proliferation and migration of non-immune cells such as epithelial cells and fibroblasts, repair the extracellular matrix (ECM), and thus promote skin repair (118). In addition, IL-22 is involved in maintaining skin homeostasis and is closely associated with the onset of diabetes. Studies have shown that mice lacking the IL-22 receptor are prone to metabolic disorders and reduced mucosal immunity under a high-fat diet. On the contrary, the administration of exogenous IL-22 can improve many metabolic symptoms, such as hyperglycemia and insulin resistance. Moreover, IL-22-Fc treatment can significantly reduce the serum lipopolysaccharide concentration and the expression of pro-inflammatory genes in adipose tissue, alleviating chronic inflammation (119, 120). Other studies have also pointed out that IL-22R1 is expressed on pancreatic islet β-cells and α-cells and may regulate the function of pancreatic islet cells through the IL-22 signaling pathway (121). Thus, the changes in the expression of IL-22 and IL-22R1 in the skin may be associated with the formation of DM ulcers and the mechanism of poor wound healing after wound infection. However, the specific signaling pathways and factors still need to be further studied and confirmed.

### 3.4 Diabetic foot, procalcitonin and C-reactive protein

Procalcitonin is a polypeptide secreted by thyroid C cells and parenchymal cells of the liver, lungs, and kidneys. It is an effective biomarker for diagnosing bacterial infections. Plasma C-reactive protein (CRP) is produced in hepatocytes and is an important indicator of the general inflammatory response. Due to tissue injury or infection, the plasma CRP concentration can increase rapidly by more than 1,000 times.

#### 3.4.1 Diagnostic role of procalcitonin

Some studies have found that infection is one of the main complications of diabetic foot ulcers and is also the main cause of amputation. When the clinical symptoms of patients are not obvious, procalcitonin can be used as an inflammatory biomarker for the diagnosis of diabetic foot ulcer infections. The researchers enrolled 15 patients with infected diabetic foot ulcers (IDFU) and compared them with 15 patients with non-infected diabetic foot ulcers (NIDFU). The results showed that the PCT level had a high efficiency in distinguishing IDFU from NIDFU, followed by C-reactive protein (CRP), white blood cell count (WBC), and erythrocyte sedimentation rate (ESR) (84). This high diagnostic accuracy was corroborated by a cross-sectional study of 185 subjects with type 2 diabetes, where PCT yielded an exceptional area under the curve (AUC) of 0.99 (95% CI, 0.96 - 1.0) for identifying IDFU, surpassing CRP (AUC 0.78), WBC (AUC 0.76), and ESR (AUC 0.74) (122). However, its utility may be limited by sensitivity. A Malaysian cross-sectional study reported that while PCT levels were significantly higher in IDFU (median 0.355 ng/mL) than in NIDFU (0.077 ng/mL) or diabetic controls (0.028 ng/mL), its sensitivity was only 63.6% at a cut-off of 0.25 ng/mL, suggesting limited value for early diagnosis despite high specificity (83.2%) (123).

#### 3.4.2 Diagnostic role of C-reactive protein and high-sensitivity CRP

In contrast, other studies position CRP as a superior biomarker. Pınar Korkmaz et al. selected 119 patients over 18 years old who were diagnosed with type 2 diabetes and diabetic foot ulcers. The results showed that compared with NIDFU cases, the levels of WBC, ESR, CRP, IL-6, and fibrinogen in IDFU cases were significantly higher (p < 0.01). The area under the ROC curve (AUROC) value of CRP was the highest (0.998; p < 0.001), and the optimal cut-off value of CRP was 28 mg/L. The optimal cut-off values of fibrinogen, IL-6, ESR, and WBC were 480 mg/dL, 105.8 pg/mL, 31 mm/h, and 11.6 (10³ μ/L), respectively. No significant effect of serum PCT level in distinguishing IDFU from NIDFU was found. The researchers believed that CRP had the highest discriminatory power in distinguishing IDFU from NIDFU (84). CRP’s value extends beyond mere diagnosis to grading infection severity. One study of 123 IDFU cases found that only serum CRP level could effectively grade the severity of the infection (124). The robustness of CRP is further supported by a meta-analysis of 7 studies, which confirmed a strong association between elevated serum CRP levels and IDFU (125).

#### 3.4.3 Comparative and combined use of PCT and CRP

The diagnostic strategy often benefits from a combined approach. In the study conducted by Jeandrot et al., when evaluating the roles of serum CRP and PCT levels in distinguishing mild infections from non-infected diabetic foot ulcers, the combination of CRP and PCT obtained the highest AUC value (AUROC: 0.947) (126) (**Table 3**). The meta-analysis by Zhang et al. concluded that both biomarkers have distinct advantages: CRP excels in distinguishing the degree of infection, while PCT holds high accuracy for diagnosing bacterial infections (125). The results of a cross-sectional study at the Hospital of the Universiti Sains Malaysia showed that the PCT level in IDFU was higher than that in NIDFU and diabetic patients, with medians (IQR) of 0.355 (0.63) ng/mL, 0.077 (0.15) ng/mL, and 0.028 (0.02) ng/mL, respectively. PCT and CRP showed a moderate positive correlation in IDFU patients (p < 0.001). The sensitivity and specificity were 63.6% and 83.2% respectively, and the optimal cut-off value was 0.25 ng/mL (126).

**Table 3:** Summary of key studies on PCT and CRP for diagnosing infected diabetic foot ulcers (IDFU).

Study (citation)	Sample size	AUC	Optimal cut-off	Sensitivity/specificity
(84)	119	0.998	28 mg/L	100%/97.37%
(122)	185	0.99	0.5ng/ml	54%/100%
(123)	264	0.79	0.25 ng/mL	63.6%83.2%
(124)	123	Distinguish between simple IDFU and IDFU:grade2 and grade 3:PCT AUC = 0.578,P=0.254,CRP AUC = 0.694,P=0.002;grade3 and grade 4:PCT AUC = 0.901;CRP AUC = 0.917,P>0.05.Distinguish between simple IDFU and IDFU complicated with other infections (IDFU+O):PCT AUC = 0.869,P<0.001.	Distinguish between simple IDFU and IDFU+O:PCT 0.59ng/ml	94.7%/88.5%
(126)	195	0.947	17mg/L	72.7%/100%

Key findings: CRP has the highest discriminatory power (84). PCT had the highest AUC for diagnosing IDFU (122). PCT level in IDFU was significantly higher than in NIDFU and controls; positively correlated with CRP and WBC (123). Combination of CRP and PCT yielded the highest AUC for distinguishing mild infections from NIDFU (124). CRP was the most informative for differentiating grade 1 from grade 2 ulcers (126).

#### 3.4.4 Limitations and future directions

Determining the definitive diagnostic superiority of PCT or CRP remains challenging due to the limited number of studies, regional variations in detection methods, and heterogeneity in study populations. The precise pathophysiological roles of CRP and PCT in the diabetic foot inflammatory process are not yet fully elucidated. Therefore, more high-quality, multi-center studies are needed to standardize protocols, validate optimal cut-off values, and clarify their mechanisms to guide precise clinical application.

### 3.5 Diabetic foot, matrix metalloproteinase-8 and matrix metalloproteinase-9

Gooyit et al. used affinity resin to identify two proteases from the wounds of diabetic mice, namely matrix metalloproteinase-8 (MMP-8) and matrix metalloproteinase-9 (MMP-9). MMP-8 is beneficial for wound repair, while MMP-9 hinders wound healing in diabetic patients. The content of active MMP-9 in the wounds of diabetic mice is 25% higher than that in non-diabetic mice (127). MMPs are involved in the immune response to infection and play an important role in the inflammatory response and healing process of wounds. After injury, neutrophils (a type of white blood cell crucial for the immune system response) arrive at the wound and secrete matrix metalloproteinase MMP-8, which is used for wound debridement (removing dead, necrotic, or infected tissue to aid wound healing) and cleaving damaged type I collagen (128). Neutrophils also secrete reactive oxygen species (ROS) to kill bacteria and regulate the formation of thrombi (blood clots) to stop bleeding. The production of ROS requires molecular oxygen. The consumption of oxygen and the destruction of blood vessels in the wound lead to hypoxia, thus delaying wound healing. T cell activation can induce the expression of MMP-2 and MMP-9, and T cell migration is mediated by MMP-9 (129). During inflammation, prostaglandin E2 upregulates the expression of MMP-9, inducing the migration of dendritic cells and thus triggering an immune response (130). In addition, MMPs are also involved in chemokine activity during the inflammatory process. For example, MMP-9 cleaves interleukin IL-8, leading to neutrophil activation. It can also cleave chemokine GRO-α (CXCL1) and CXCL4 (platelet factor-4) (131). The level of NF-κB increases with the severity of the wound and the degree of infection and is closely related to active MMP-9. Some researchers have used MMP-9 knockout mice with streptozotocin-induced diabetes to confirm the role of MMP-9. Streptozotocin destroys the insulin-producing β cells in the pancreas. MMP-9 gene ablation promotes diabetic wound healing, thus confirming the harmful effect of MMP-9 (132). After infecting diabetic mice with Staphylococcus epidermidis, some researchers found that there was no statistically significant change in the level of active MMP-8. By the 7th day, the active MMP-9 in the wounds of infected diabetic mice was 20% higher than that in non-infected diabetic mice (133). In addition, infection increases active MMP-9, exacerbates inflammation, and inhibits angiogenesis. Therefore, compared with non-infected wounds, infected diabetic wounds take longer to heal. The best strategy for treating diabetic foot ulcers (DFUs) is to selectively inhibit the harmful protease MMP-9 without affecting the beneficial MMP-8, enabling the body to repair the wound (116). Based on this, the combined treatment with a selective MMP-9 inhibitor and exogenous MMP-8 may have better efficacy for wound healing in diabetic patients, that is, the use of protease-antiprotease combination therapy. ND-336 is a highly selective MMP-9 inhibitor, and the presence of MMP-8 can promote diabetic wound healing (133). Therefore, the use of ND-336 and exogenous MMP-8 can greatly promote the recovery of wounds or ulcers in diabetic patients, such as diabetic foot. The efficacy of the two has been confirmed in diabetic mice, but there is a lack of evidence from relevant clinical trials.

### 3.6 Diabetic foot and neutrophil-to-lymphocyte ratio

The NLR index is a major component of the chronic inflammatory response. In this regard, an increased proportion of neutrophils indicates an active inflammatory process, while a decreased proportion of lymphocytes reflects an insufficient immune response, which is one of the characteristics of type 2 diabetes (134).

#### 3.6.1 NLR as a predictor of ulcer healing and prognosis

NLR is a significant predictor of healing outcomes in diabetic foot ulcers (**Table 4**). A prospective study established a strong association between NLR values and healing status. Patients with an NLR less than 6 had a 75.9% healing rate, whereas ulcers in all patients (100%) with an NLR greater than 6 failed to heal. The mean NLR was significantly lower in the healing group (5.15) compared to the non-healing group (8.205). With a sensitivity of 100% and a negative predictive value of 100%, NLR proves to be an excellent screening tool to identify patients at high risk for non-healing and poor prognosis (135). Recent studies have shown that NLR values are higher in patients with peripheral neuropathy and foot ulcers compared to those without diabetic foot (134, 136, 137). Vatankhah et al. (138) further confirmed that a higher NLR is associated with a higher incidence of non-healing ulcers.

**Table 4:** Summary of key studies on the prognostic and diagnostic value of NLR in diabetic foot.

Outcome measured	Study (citation)	Key finding	NLR cut-off/value	Statistical performance
Ulcer Healing	(135)	NLR >6 associated with 100% non-healing rate.	6	Sensitivity: 100%; Specificity: 75.9%;
Ulcer Healing	(138)	Higher NLR associated with a higher incidence of non-healing ulcers.	4.19	Sensitivity: 63%; Specificity: 71%;AUC=0.68
All-Cause Mortality of people with diabetic foot ulcer (Post-Amputation)	(139)	High NLR group had significantly lower 1, 3, and 5-year survival rates.	2.76	Sensitivity: 69.2%; Specificity: 62.6%;AUC=0.679
Sepsis Prediction	(140)	NLR is an independent prognostic factor for sepsis, with better predictive value than CRP.	6.814	Sensitivity: 75%; Specificity: 82.5%;AUC: 0.790 (NLR) vs 0.780 (CRP)
Correlation with Oxidative Stress	(142)	NLR positively correlated with oxidative stress index in DF patients.	1.9	Sensitivity: 78.3%; Specificity: 64.7%;AUC=0.760

#### 3.6.2 Association with mortality and severe complications

The prognostic value of NLR extends to predicting long-term survival after major adverse events. NLR is significantly associated with all-cause mortality following amputation. Patients in a low NLR group had markedly higher overall survival rates at 1 (96.8%), 3 (84%), and 5 years (80.1%) post-amputation. In stark contrast, the high NLR group had survival rates of 85.2%, 58.6%, and 23.9% at the same time points (p < 0.001) (139). Furthermore, NLR demonstrates superior predictive value for severe complications like sepsis compared to traditional markers. The researchers divided 216 patients with DFU into a sepsis group and a non-sepsis group and performed a multivariate logistic regression analysis. The results suggested that NLR and CRP were independent prognostic factors for sepsis caused by DFU. Receiver operating characteristic (ROC) curve analysis showed that the area under the curve (AUC) of NLR was 0.790, significantly higher than 0.780 of CRP, indicating that the predictive value of NLR was better than that of CRP (140).

#### 3.6.3 Comparative value with other inflammatory markers

The diagnostic utility of NLR must be contextualized alongside other biomarkers. While NLR is a powerful prognostic tool, its efficacy for specific diagnoses like osteomyelitis (OM) is more limited. One study comparing NLR, platelet-to-lymphocyte ratio (PLR), and CRP found that CRP and erythrocyte sedimentation rate (ESR) were most elevated in severe (Grade 3) DFU and were superior for distinguishing Wagner grades and detecting OM. Although NLR and PLR correlated with CRP, ESR, and DFU grade, their ROC performance for distinguishing DFU patients or diagnosing OM was poor compared to CRP (141).

#### 3.6.4 Pathophysiological correlations

The elevation of NLR in DFU is intertwined with other hematological and pathophysiological changes. Studies have observed that DFU patients often present with anemia, as evidenced by significantly decreased hemoglobin levels and red blood cell counts (138). This anemia of chronic disease, coupled with a significantly higher prevalence of anemia in DFU groups (60.9% vs. 20.3% in non-DFU), contributes to tissue hypoxia and impaired healing. The altered WBC subset distribution—increased neutrophils and decreased lymphocytes—directly drives the NLR increase and is mechanistically linked to oxidative stress, as evidenced by a significant positive correlation between NLR and an oxidative stress index in DFU patients (r = 0.449; P < 0.05) (142).

In summary, NLR is a readily available, cost-effective, and powerful prognostic biomarker for predicting diabetic foot ulcer healing, amputation mortality, and severe infections like sepsis. Its strength lies in reflecting the overall inflammatory-immune balance. However, it may be less effective than CRP or ESR for specific diagnoses like osteomyelitis. Therefore, NLR should be interpreted as part of a comprehensive clinical assessment alongside other biomarkers and clinical findings.

## 4 Treatments of diabetic foot

The treatment of diabetic foot has always been a major challenge. Due to the peripheral neuropathy and decreased sensory function of the feet in patients with diabetic foot, early lesions are difficult to detect. In most cases, the foot lesions are already very severe by the time of diagnosis, often requiring amputation. In the past, the treatment of diabetic foot focused on the improvement of surgical methods. However, at present, limb salvage surgery and revascularization surgery are not yet mature, and there are numerous postoperative complications in patients with diabetic foot, resulting in a poor quality of life.

Some studies have shown that there are many postoperative complications of ankle and foot tendon surgery, including but not limited to infection, difficult wound healing, wound dehiscence, sural neuritis, fistula, scar adhesion, deep vein thrombosis, etc. The risk of postoperative complications in diabetic patients increases, especially the difficulty in wound healing (143, 144). A retrospective analysis of patients with diabetic foot ulcers after surgery showed that among 129 diabetic patients, 56% of the patients needed additional surgery within 12 months after the first surgery, and 30% of the patients needed amputation. In particular, the proportion of patients who needed additional surgery after surgical debridement was very high (75%) (17).

The occurrence of the above complications and repeated surgeries undoubtedly cause secondary harm to diabetic patients and greatly increase the economic burden on patients. If in-depth exploration can be carried out based on the pathological mechanism of diabetic foot and the molecular pathways related to the inflammatory response, it may provide a theoretical basis for optimizing clinical intervention strategies, thereby effectively relieving the pain symptoms of patients, significantly improving the quality of life, and delaying the progression of diabetic foot ulcers to the clinical outcome of amputation.

Studies have shown that salicylates can improve blood glucose control and insulin sensitivity in patients with type 2 diabetes mellitus (T2DM) by inhibiting the activation of NF-κB (145) (**Table 5**). Treating T2DM patients with salicylates (a prodrug of salicylic acid) targeting the NF-κB pathway has beneficial effects on blood glucose, triglyceride, free fatty acid (FFA), and C-reactive protein (CRP) concentrations and can improve glucose utilization (146). A randomized, placebo-controlled, parallel-phase 3 clinical trial further confirmed that after 48 weeks of salicylic acid treatment, blood glucose was significantly improved and inflammatory mediators were reduced in T2DM patients (147). The improvement of blood glucose and the reduction of inflammatory mediators are both beneficial for the healing of diabetic foot wounds. Recently, some researchers have found that lactoferrin can reduce diabetes-related inflammation and is also related to the NF-κB pathway. Molecular dynamics simulations show that lactoferrin has the strongest interaction with the B chain of NF-κB, and the RMSD trajectory shows a high degree of consistency between the two during the simulation. Lactoferrin can interact with six major components of the NF-κB signaling pathway (IL-1β, IL-6, IκBα, NF-κB, IKK, and TNF-α). Among them, the interactions with IL-1β, IL-6, IκBα, and NF-κB are relatively stable, while the interactions with IKK and TNF-α are relatively weak. Through its interaction with the NF-κB signaling pathway, lactoferrin can inhibit the activation of this pathway and reduce the production of pro-inflammatory cytokines, thus having potential preventive and therapeutic applications in diabetes management (148).

**Table 5:** The treatment methods of diabetic foot involved in this article.

Treatment	Possible involved inflammatory mechanisms	References
Salicylates	Inhibit the activation of NF-κB	(145–147)
Elebela	Downregulate serum inflammatory factors, inhibit TRAF1, and suppress NF-κB signaling pathway	(149, 150)
Lactoferrin	Inhibit the activation of NF-κB	(148)
Dimethyl fumarate	Inhibit the activation of NF-Kb and oxidative stress	(151)
Betulinic acid	Alleviate hyperglycemia-induced oxidative stress and inflammation through Nrf2 activation and NFκB p65 signaling inhibition	(152)
Huangbai liniment	Reduce oxidative stress, increase the level of TGF-β1, and decrease the level of MMP9	(153)
Inosine	Inhibit NF-κB p65 and enhance Nrf2 transcriptional activity, suppress the AGE/RAGE axis, and alleviate oxidative stress	(154)
25-OH-vitamin D	unclear	(155, 156)
the NO microbubble-captured hydrogel	Reduce the levels of pro-inflammatory cytokines (IL-1β, IL-6, and TNF-α) and increase the levels of anti-inflammatory cytokines (IL-10, IL-22, and IL-13)	(157)
Hyperbaric oxygen	Maintain the balance between pro-inflammation and anti-inflammation	(63)
(R)-ND-336	Inhibit MMP-9, suppress the NF-κB pathway, and alleviate oxidative stress	(133)

Elabela (ELA) is the second ligand of the apelin receptor (APJ) and is expressed only in specific tissues, including the heart, kidneys, endothelium, and blood vessels. ELA is generated by the proteolysis of its precursor peptide ELA-54, which contains 54 amino acids and has been shown to promote angiogenesis and the development of the cardiovascular endothelial system (149). Some researchers applied ELA to the area around the wounds of db/db mice and found that it could reduce inflammatory infiltration, promote wound healing, and significantly downregulate the expression of serum inflammatory cytokines and 8-hydroxyguanosine. Moreover, ELA can downregulate NF-κB signaling by inhibiting TRAF1, reduce DNA damage induced by reactive oxygen species, promote endothelial cell migration and angiogenesis, thus being beneficial for the healing of diabetic feet (150).

Some studies have found that nuclear factor erythroid 2-related factor 2 (Nrf2) plays a positive role in the treatment and recovery of diabetic foot ulcers. The Nrf2 activator dimethyl fumarate (DMF) can improve oxidative stress and inflammation mediated by Nrf2 inhibition and accelerate the healing of diabetic wounds (151). Researchers intervened in the wounds of diabetic rats with dimethyl fumarate. The results showed that overexpression of Nrf2 or DMF treatment significantly reduced the generation of reactive oxygen species caused by high glucose treatment, while knockdown of Nrf2 increased the generation of reactive oxygen species; overexpression of Nrf2 or DMF treatment reduced the secretion of IL1β, IL6, and MCP1 caused by high glucose treatment, while knockdown of Nrf2 increased the secretion of these cytokines. As an Nrf2 activator, DMF significantly accelerated wound healing in the diabetic rat model, reducing oxidative stress and the expression of pro-inflammatory cytokines. This provides a new clinical treatment strategy for diabetic wound healing.

Similar to the mechanism of action of dimethyl fumarate and beneficial for diabetic wounds are betulinic acid, theaflavin, and huangbai liniment. Betulinic acid improves diabetes-mediated glucose tolerance by activating the expression of glucose transporter 4 (GLUT4), reduces oxidative stress and inflammation induced by hyperglycemia through Nrf2 activation and NFκB p65 signal inhibition, and exerts a vasoprotective effect by stimulating the expression of endothelial nitric oxide synthase. Betulinic acid significantly accelerated the healing of diabetic rat wounds through two methods: intraperitoneal injection and local administration, indicating that it may be a promising therapeutic candidate for the treatment of diabetic wound healing (152). Huangbai Liniment has an obvious protective effect on diabetic wound healing. Reducing oxidative stress may be the key factor in wound healing mediated by Huangbai Liniment. RNA-seq analysis and further experiments show that the activation of Nrf2 and its downstream antioxidant genes reduces apoptosis and oxidative damage, thus improving the wound healing mediated by Huangbai Liniment. HB Liniment also increases the level of TGF-β1 and decreases the level of MMP9, thereby promoting diabetic wound healing (153).

Another study found that inosine can alleviate diabetic peripheral neuropathy. As previously mentioned, diabetic peripheral neuropathy is also an important factor that exacerbates diabetic foot. The study found that inosine can be used to treat peripheral neuropathy in hyperglycemic-induced rats. It reduces blood glucose levels without affecting body weight, enhances glyoxalase 1 (GLO1), reduces oxidative stress by inhibiting the AGE/RAGE axis, and thus reduces NF-κB p65 and its phosphorylated form. On the other hand, inosine upregulates Nrf2 and heme oxygenase-1 (HO-1), sequentially increases antioxidant molecules such as superoxide dismutase and catalase, and simultaneously reduces lipid peroxidation. In addition, it relieves pain by downregulating the expression of PKC and TRPV1, reducing substance P, and decreasing TGF-β, and is expected to be a candidate drug for the treatment of diabetic foot (154).

In recent years, the correlation between vitamin D and diabetes has received extensive attention. A retrospective analysis shows that vitamin D deficiency is prevalent in patients with diabetic foot ulcer (DFU). Vitamin D deficiency (< 50 nmol/L) accounts for 86.78% of all DFU patients, and the vitamin D level in DFU patients is lower than that in diabetes mellitus (DM) patients. The difference is statistically significant (p < 0.05) (155). Another randomized double-blind clinical trial found that the ulcer healing rate of diabetic patients in the high-dose vitamin D3 group was 70%, which was significantly higher than 35% in the low-dose group. The median ulcer area in the high-dose group decreased by 100%, while that in the low-dose group was 57%, indicating that high-dose vitamin D3 has significant clinical effects in promoting the healing of chronic diabetic foot ulcers. However, the specific mechanism is still unclear and requires further verification by basic experiments (156).

In addition, some researchers have successfully developed a novel *in-situ* hydrogel system by using the characteristics of nitric oxide to promote angiogenesis and antibacterial activity. This system accelerates the healing of diabetic foot by capturing NO microbubbles. It can reduce the levels of pro-inflammatory cytokines (IL-1β, IL-6, and TNF-α) and increase the levels of anti-inflammatory cytokines (IL-10, IL-22, and IL-13), bringing new treatment options for diabetic foot patients (157).

## 5 Discussion

The pathological development process of diabetic foot involves the interaction of multiple mechanisms: Neuropathy leads to the loss of protective sensation, reducing the patient’s ability to perceive mechanical stress. Vascular lesions result in insufficient tissue perfusion, thereby creating a hypoxic and ischemic microenvironment. Immune dysfunction causes an imbalance between pro-inflammation and anti-inflammation, further increasing the risk of infection.

In the past, most studies on the pathogenesis of diabetic foot focused on diabetic neuropathy. It was generally believed that diabetic foot ulcers were the result of multiple diabetic neuropathies. Specifically, sensory neuropathy leads to the loss of protective sensation, motor neuropathy causes foot deformities and biomechanical abnormalities, and autonomic neuropathy results in skin changes. On this basis, factors such as minor trauma, inflammation, continuous low or high pressure during weight-bearing in patients can all lead to the appearance of ulcers and may also cause bleeding under the callus (158).

In recent years, the research field of diabetic neuropathy has evolved from a simple glucose-centered view to a more comprehensive understanding. Diabetic neuropathy (DN) is a complex disorder secondary to multiple related metabolic and inflammatory injuries. As a key pathological link, the inflammatory response forms a vicious cycle of “inflammation-oxidative stress-tissue damage” through signal pathways such as NF-κB and the IL family. The high-glucose environment can activate NF-κB through the AGEs-RAGE axis, upregulate the expression of pro-inflammatory factors such as IL-6 and TNF-α, and simultaneously inhibit the secretion of anti-inflammatory mediators such as IL-10, ultimately leading to an imbalance in the inflammatory homeostasis (5, 6). This dynamic imbalance between pro-inflammatory and anti-inflammatory factors directly affects the wound healing process. The imbalance of inflammatory regulation further increases the risk of infection in diabetic foot ulcers, and infection and progressive gangrene are the main reasons for lower limb amputation in patients with diabetic foot (7).

In the classification system of diabetic foot, Charcot foot has received extensive attention due to its unique mechanism of bone and joint destruction. Charcot foot is a rare but highly disabling complication caused by diabetic neuropathy. Epidemiological data show that its prevalence in diabetic patients is 0.56% (8). This disease is essentially a neurogenic osteoarthropathy, and its typical manifestations are progressive bone destruction and deformities in the ankle and foot (such as the “rocker-bottom” deformity) (9). Its pathological mechanism involves a triple interaction. Peripheral/autonomic neuropathy leads to the loss of protective sensation and vasomotor dysfunction. Local trauma triggers a cascade release of pro-inflammatory factors such as IL-1β, IL-6, and NF-κB, activates the RANKL/RANK/OPG signal axis, and increases the activity of osteoclasts compared to normal conditions. The deficiency of anti-inflammatory neurotransmitters (such as CGRP, SP, etc.) leads to an imbalance in bone metabolism, and the serum RANKL/OPG ratio is higher than that of healthy people (159), thus accelerating bone resorption.

The disease process of Charcot foot is divided into the acute phase (local temperature increase of 2 - 8 °C, painless swelling) (22, 23) and the chronic phase (bone deformity). Among them, the symptoms of the acute phase are easily overlooked, making it difficult to diagnose (24). If not treated in a timely manner, it may progress to irreversible structural damage.

In recent years, studies have found that Denosumab can reduce the activity of osteoclasts by targeting and inhibiting RANKL (31). There are case reports suggesting that it can shorten the course of the acute phase (32), but currently, there is a lack of evidence from multi-center randomized controlled trials (RCTs). It should be noted that although patients with diabetic neuropathy generally have reduced bone density (160), only 0.56% of patients will develop Charcot foot, which may be related to NO-mediated vasodilation disorders. Under diabetic conditions, the synthesis of NO is reduced, which enhances the activity of vascular matrix metalloproteinases (35), limiting the local spread of inflammation. This special “nerve-inflammation-vessel” interactive network provides new targets for precise intervention.

Currently, the clinical treatment of diabetic foot faces two major challenges. On the one hand, traditional surgical interventions (such as debridement and revascularization) have a relatively high incidence of postoperative complications. Clinical studies have shown that the proportion of patients with diabetic foot who need reoperation after surgical debridement is as high as 75% (17). A meta-analysis shows that the success rate of non-surgical treatment for diabetes-related foot osteomyelitis is 68.2%, while the success rate of surgical treatment is 85.7% (18). Although the success rate of non-surgical treatment is relatively low, considering that surgery may deteriorate the patient’s physical condition, in some cases, non-surgical treatment may be a more appropriate choice. On the other hand, targeted therapies for inflammatory regulation are still in the exploratory stage. Basic research shows that regulating the NF-κB pathway is helpful for improving wound healing. For example, salicylates can reduce the level of IL-6 by inhibiting the phosphorylation of IKKβ (145), and lactoferrin can prevent NF-κB from entering the nucleus through a steric hindrance effect (148), etc. These research results provide a theoretical basis for the development of new anti-inflammatory therapies, but the identification and application of key regulatory nodes still require further research.

Systemic anti-inflammatory agents exhibit insufficient tissue selectivity, and this limitation may be overcome by advanced local delivery systems (e.g., nanocarriers or hydrogels). These systems enable the targeted release of small interfering RNA (siRNA) or gene therapeutics, thereby achieving spatiotemporal control of inflammation at the wound site. Smart responsive hydrogels improve the wound microenvironment by dynamically regulating drug release, humidity, temperature, and pH. They reduce drug resistance and secondary injury, enhance patient compliance, and thus provide a novel strategy for the treatment of diabetic wounds (161). For instance, matrix metalloproteinase-2 (MMP-2)-responsive hydrogels degrade via MMP-sensitive peptide chains, enabling the sustained release of adipose-derived mesenchymal stem cell exosomes (ADSC-exo) for up to 20 days, which promotes epithelial regeneration and angiogenesis. Glucose-responsive hydrogels containing phenylboronic acid (PBA) groups can release insulin when blood glucose levels rise, thereby reducing inflammation and promoting collagen deposition. pH-responsive hydrogels loaded with silver nanoparticles (AgNPs) rapidly release Ag^+^ under acidic conditions (e.g., in infected wounds) to kill bacteria and facilitate angiogenesis. Additionally, hydrogels containing tannic acid (TA) scavenge reactive oxygen species (ROS) through the cleavage of borate ester bonds or disulfide bonds, inhibit the infiltration of M1-type macrophages, and shorten the inflammatory phase.

This article systematically reviews the roles of key inflammatory factors such as NF-κB and the IL family and potential therapeutic targets. Although significant progress has been made in recent years, clinical translation still faces many challenges and needs to be further explored from the following perspectives.

### 5.1 The dynamic balance of the inflammatory network and clinical paradoxes

#### 5.1.1 Spatiotemporal regulation of pro-inflammatory and anti-inflammatory factors

IL-6 may have dual roles in inflammation and angiogenesis. High concentrations of IL-6 exacerbate bone destruction (such as Charcot foot) through the JAK-STAT pathway (85, 87). However, other studies have shown that pharmacological doses of IL-6 can accelerate the motor and sensory nerve conduction velocities in diabetic rats and promote an increase in nerve blood flow (55, 91, 92). The classical signaling pathway of IL-6 is crucial for maintaining the angiogenesis function of vascular endothelial cells (ECs). In contrast, the trans-signaling pathway of IL-6 inhibits the migration, proliferation, and tubular structure formation ability of vascular endothelial cells. When the endogenous IL-6 expressed by ECs is depleted, their ability to form tubular structures is impaired, indicating that autocrine IL-6 classical signaling is necessary for the angiogenesis function of ECs. However, at the molecular level, IL-6 trans-signaling upregulates known anti-angiogenic factors such as CXCL10 and SERPINF1 in ECs, while downregulating known pro-angiogenic factors such as cKIT and CXCL8. In addition, IL-6 trans-signaling also alters the response of ECs to vascular endothelial growth factor-A (VEGF-A), resulting in increased p38 phosphorylation and decreased Erk1/2 phosphorylation (95). Similarly, IL-1β drives inflammation through the NETs-NLRP3 axis in the acute phase (67), but insufficient regulation of it delays wound healing (69). The disruption of this dynamic balance may explain the chronic tendency of diabetic foot ulcers, and it is necessary to develop stratified intervention strategies based on the inflammatory stage.

#### 5.1.2 Heterogeneous controversies of diagnostic markers and the microbiome-immune interface

There are contradictions in the effectiveness of CRP and PCT in the diagnosis of infectious diabetic foot ulcers (IDFU). The broad inflammatory sensitivity of CRP gives it an advantage in distinguishing the degree of infection (AUROC 0.998) (84), while PCT has a higher specificity for bacterial infection (cut-off value 0.25 ng/mL) (123).

Furthermore, the dysbiotic wound microbiome perpetuates chronic inflammation and impairs healing, representing a key component of the inflammatory microenvironment. Pathogenic bacteria (e.g., Staphylococcus aureus) initiate infection by attaching to host cells via adhesins expressed on their surface (162). They fulfill their basic nutritional requirements by locally disrupting red blood cells and releasing iron (163). Additionally, bacteria can use adhesins to invade osteoblasts and fibroblasts, leading to non-healing of diabetic foot ulcers and an increased risk of amputation (164). Adhesin antibodies can significantly inhibit the attachment of bacteria to target tissues, prevent biofilm formation, and block the initiation of infection (165). Microbial communities form biofilms by embedding themselves in a mucous extracellular polymeric matrix, which prevents the penetration of host immune defenses and antimicrobial therapies (166). The high bacterial population within biofilms increases the rate of horizontal gene transfer (HGT) among biofilm residents, creating a rich pool of traits. This ultimately leads to the natural selection of virulence genes and antimicrobial resistance genes (167), resulting in recurrent diabetic foot ulcers. Furthermore, immunoglobulin proteases can disrupt the humoral components of the body’s immune defense (IgA, IgG, IgM), enabling pathogens to evade the host’s immune response (168) and thus causing prolonged wound healing. Future research needs to combine multi-omics technologies (such as exosomal miRNAs and metabolomics) with artificial intelligence (AI) modeling to establish a combined diagnostic model to improve prediction accuracy. For example, Zhang et al. employed techniques such as single-cell sequencing, immunogenic cell death (ICD) scoring, cell communication analysis, and Mendelian randomization to demonstrate that ectonucleoside triphosphate diphosphohydrolase 1 (ENTPD1) promotes diabetic foot ulcer healing by regulating ICD levels and cell communication in Vasendo. Genes such as ankyrin B1 (ANKB1) and acidic leucine-rich nuclear phosphoprotein 32 family member E (ANP32E) may mediate the occurrence of diabetic peripheral arterial disease (DPAD) by affecting Vasendo function. ENTPD1^+^ Vasendo and related pathways (e.g., atypical chemokine receptor 1 (ACKR1), macrophage migration inhibitory factor (MIF)) may serve as therapeutic targets for DFUs and DPAD (169).

### 5.2 Opportunities and challenges of targeted therapy and regenerative therapy

#### 5.2.1 The translational potential of NF-κB pathway regulation

Salicylates and lactoferrin reduce the levels of IL-6/TNF-α by inhibiting IKKβ phosphorylation or blocking NF-κB nuclear translocation (145, 148). However, the insufficient tissue selectivity may affect the therapeutic effect. Local delivery systems (such as nanocarriers) can optimize drug targeting and reduce systemic side effects. In addition, Nrf2 activators (such as DMF and betulinic acid) synergistically regulate NF-κB by inhibiting oxidative stress, providing new ideas for multi-pathway combined interventions (151, 152).

#### 5.2.2 Bidirectional regulation strategies of the IL family

The wound-healing promoting effect of IL-10 has been verified in mouse models (105), but its expression in human diabetic feet is affected by factors such as microenvironmental oxygen tension and microbiota. Monoclonal antibody therapy targeting the IL-17/IL-23 axis can accelerate re-epithelialization (113), but attention should be paid to the risk of infection caused by immunosuppression. The angiogenesis-promoting and metabolic regulatory functions of IL-22 (118, 119) suggest that it may become a cross-mechanism therapeutic target, but the problem of heterogeneous receptor expression needs to be solved.

#### 5.2.3 Clinical implications of bone-immune interaction and regenerative approaches

The imbalance of RANKL/OPG in Charcot foot is closely related to the activation of the IL-6/JAK2 signal (10, 87). Although Denosumab can inhibit bone resorption in the short term (31, 32), long-term use may exacerbate vascular calcification. Combined anti-inflammatory therapy (such as local delivery of IL-4) may break the vicious cycle of “inflammation-bone destruction”, and its safety needs to be verified through multi-center RCTs.

Beyond pharmacological approaches, regenerative medicine strategies, such as mesenchymal stem cell (MSC)-derived exosomes and tissue-engineered scaffolds, have emerged as promising avenues for modulating the hostile inflammatory microenvironment, promoting angiogenesis, and facilitating tissue repair in DFU. Mesenchymal stem cells (MSCs) can alleviate the post-injury inflammatory microenvironment in the bone marrow and prevent excessive activation and exhaustion of hematopoietic stem cells (HSCs) by secreting anti-inflammatory factors such as TGF-β and interleukin-10 (IL-10). They can also induce angiogenesis; by regulating immune responses and inflammation, MSCs ultimately improve local blood flow to damaged perfused and circulatory tissues, thereby reducing the severity of diabetic foot ulcers (DFUs) and the amputation rate (170, 171). Mesenchymal stem cell-derived exosomal microRNA-21-5p (miRNA-21-5p) is a key molecule that promotes ischemic repair and angiogenesis in diabetic feet. It upregulates vascular endothelial growth factor receptors (VEGFRs) and activates serine/threonine kinases (e.g., Akt) and mitogen-activated protein kinases (MAPKs), thereby facilitating angiogenesis (172). Traditional dressings (e.g., hydrogels, lyophilized scaffolds) cannot replicate the fibrous network of the extracellular matrix (ECM). In contrast, electrospun nanofibers (ENs) have emerged as ideal materials for wound healing and tissue engineering due to their high surface area-to-volume ratio, porous interconnected structure, and biomimetic topological morphology. Multifunctional electrospun nanofibers (MENs) further integrate functions such as drug delivery, antibacterial activity, and pro-angiogenic properties, providing a cell-free therapeutic strategy for chronic wounds (e.g., diabetic foot ulcers) (173). Recently, nanomaterials, as drug carriers, have attracted widespread attention due to their ability to improve drug bioavailability and maintain stable drug release (174). Biomimetic drug delivery systems are designed by combining cell membrane-coated nanoparticles with synthetic nanoparticles, enabling them to replicate the key functional characteristics of native cells (175).

### 5.3 Optimization directions of clinical practice towards precision medicine

#### 5.3.1 Construction of an individualized treatment framework

Based on prognostic markers such as NLR and IL-10 (135, 138), a risk stratification system for diabetic feet can be established. Low-risk group (NLR < 6): Non-surgical treatment [negative pressure therapy, anti-inflammatory drugs] is preferred. High-risk group (NLR ≥ 6, IL-6 ≥ 20 pg/ml (85)]: Early combined revascularization and targeted anti-inflammatory therapy (such as IL-1β antagonists) are recommended.

#### 5.3.2 Integration of new treatment technologies

The *in-situ* hydrogel system regulates the release of NO to balance pro-inflammatory and anti-inflammatory factors (157). Combining 3D bioprinting technology to customize functionalized, drug-loaded scaffolds dressings may break through the limitations of traditional debridement. This approach allows for the localized and sustained delivery of immunomodulatory agents (e.g., anti-IL-6 siRNA or IL-10), directly addressing the spatial dysregulation of inflammation. In addition, the high-dose supplementation of vitamin D3 (70% healing rate) (156) indicates the auxiliary value of nutritional intervention. It is necessary to explore its molecular mechanism with inflammatory pathways (such as the interaction between VDR and NF-κB).

### 5.4 Limitations and future prospects

Most current studies are limited to animal models or small-sample clinical observations. The following directions need urgent breakthroughs. Insufficient spatiotemporal resolution in mechanism research: Single-cell sequencing can analyze the inflammatory characteristics of different cell subsets (such as M1/M2 macrophages and fibroblasts) at the ulcer edge. Tissue-specific differences in therapeutic targets: The differences in the signal transduction of IL-6 in skin and bone tissues need to be clarified through conditional gene knockout models. Bottlenecks in clinical translation: Most targeted drugs (such as ND-336 and ELA) have only completed preclinical verification (127, 150). It is necessary to promote phase I/II trials to evaluate efficacy and safety. Future research must prioritize the integration of multi-omics data (genomic, proteomic, metabolomic) with advanced AI analytics to decode the complex inflammatory networks in DFU, ultimately paving the way for truly personalized predictive diagnostics and therapeutic interventions.

## 6 Conclusions

The inflammatory regulatory network of diabetic foot shows a high degree of complexity and dynamics. Future research needs to integrate multi-omics data and artificial intelligence models to achieve precise typing and individualized treatment. By targeting the NF-κB/IL axis, optimizing the combined use strategy of biomarkers, and innovating the local drug delivery system, it is expected to break through the existing treatment bottlenecks, improve the prognosis of patients, and reduce the medical burden.
